# Development of a tongue ultrasound-based predictive model for hypoxemia during painless gastroscopy in ASA I-II patients

**DOI:** 10.7717/peerj.20634

**Published:** 2026-01-14

**Authors:** Hao Wu, Xu Chen, Guanfeng Hou, Xuebing Zhang, Wei Zhang, Sheng Wang, Lijian Chen

**Affiliations:** 1 Department of Anesthesiology, The First Affiliated Hospital of Anhui Medical University, Hefei, Anhui, China; 2 Department of Anesthesiology, The First Affiliated Hospital of USTC, Division of Life Sciences and Medicine, University of Science and Technology of China, Hefei, Anhui, China

**Keywords:** Hypoxemia, Painless gastroscopy, Nomogram, Ultrasonography

## Abstract

**Background:**

The risk of hypoxemia in painless gastroscopy has been widely recognized, but reliable predictors are still lacking. Tongue ultrasonography has been shown to facilitate the identification of difficult airways. In this study, we hypothesize that tongue ultrasonography may predict hypoxemia during painless gastroscopy, and aim to develop a predictive model for hypoxemia based on its prognostic value.

**Methods:**

This study included 304 patients underwent painless gastroscopy. Common and tongue ultrasound indicators were used for the prediction, including body mass index (BMI), Mallampati test score, tongue thickness (TT) and hyomental distance. Univariate and multivariate logistic regression were used to identify independent predictors of hypoxemia. Nomograms were constructed to predict hypoxemia based on the logistic regression analysis results and established risk factors documented in prior literature. Receiver operating characteristic (ROC) curves were used to evaluate the accuracy of the nomograms. The nomogram was internally validated.

**Results:**

BMI, Mallampati score, TT, and popofol dose were integrated for hypoxemia nomogram. The areas under the ROC curves were 0.833 (95% confidence interval (CI) [0.762–0.904]). The calibration curve and decision curve analysis of the prediction model indicated that the model could have favourable predictive ability.

**Conclusion:**

Nomograms based on tongue ultrasonography could be a reliable tool in predicting hypoxemia during painless gastroscopy.

## Introduction

Painless gastroscopy, defined as esophagogastroduodenoscopy performed under sedation, has become an integral part of standardized health management protocols. This procedure exhibits substantial clinical value in the early detection and prevention of gastrointestinal diseases (Zhang et al., 2022). Nevertheless, certain adverse reactions linked to painless gastroscopy—such as hypoxemia and reflux aspiration—continue to present significant challenges. These complications not only pose considerable risks to patients but also necessitate meticulous perioperative management by healthcare providers (Choe et al., 2024; Nonaka et al., 2015).

Hence, preoperative risk assessment for safety in painless gastroscopy remains imperative, where established predictive parameters including elevated body mass index (BMI) and Mallampati classification have been incorporated into hypoxemia risk stratification during procedural sedation (Early et al., 2018). However, current parameters exhibit discernible methodological constraints, particularly regarding the subjective variability in Mallampati classification assessment (Green & Roback, 2019). Although these parameters have been operationalized in clinical settings, their standalone predictive capacity may remain inaccurate given the multifactorial etiology of sedation-related complications.

A recent study involving 999 outpatients undergoing painless gastroscopy demonstrated that prediction models incorporating airway assessment parameters significantly improved predictive performance (Xiong et al., 2023). This finding provides compelling rationale for believing that refining the precision of airway parameter evaluations could enable the development of models with substantial predictive advantages.

The rising application of ultrasound in airway assessment has enhanced our recognition of certain anatomically critical regions that are otherwise unobservable through surface examination. Studies have demonstrated that tongue thickness measured by ultrasound can accurately and effectively predict the presence of difficult airways, with superior predictive value compared to the modified Mallampati classification (Yao & Wang, 2017). Furthermore, a research revealed that the ultrasound-measured hyomental distance is a more reliable indicator of difficult airways than the externally measured thyromental distance (Wang et al., 2022).

Despite the well-established advantages of tongue ultrasonography in airway assessment, its potential utility in predicting hypoxemia risk during painless gastroscopy remains unclear. We hypothesize that ultrasound-derived tongue metrics correlate with the occurrence of hypoxemia in painless gastroscopy and may facilitate the development of an integrated predictive model through comprehensive evaluation.

## Materials & Methods

The Medical Ethics Committee of Anhui Provincial Hospital, certificated the study protocol (Approval no. 2019KY-108). It was also registered at the China Clinical Trial Registration Center on May 16, 2020 (ChiCTR2000032940), before patient enrollment. All patients provided informed consent for the trial procedures and signed written consent forms. This study was designed and reported in accordance with the Standards for Reporting of Diagnostic Accuracy (STARD) guidelines (Bossuyt et al., 2015).

This prospective observational study was conducted from May 16, 2020 to September 4, 2021. Patients who were undergoing painless gastroscopy after assessment in the anesthesia clinic were included. The following inclusion criteria were used: (i) 18 to 80 years old patients; (ii) American Society of Anesthesiologists (ASA) score of I, II; (iii) no upper airway anatomical deformity. The following exclusion criteria were used: (i) patients who dropped out of the study; (ii) patients who temporarily changed painless gastroscopy to conventional gastroscopy due to fever, upper respiratory tract infection, respiratory tract inflammation; and (iii) patients with baseline peripheral oxygen saturation (SpO_2_) levels <95%.

Prior to painless gastroscopy, patients were admitted to the endoscopy holding area for final pre-procedural assessment and confirmation. We evaluated Mallampati classification for all participants. The Mallampati test was measured as criterion (Samsoon & Young, 1987): the patient assumed an upright seated position, opened the oral cavity widely, and protruded the tongue to the fullest extent. Scoring was then conducted based on the observable pharyngeal anatomical structures, with grading criteria defined as follows: Grade 1, visible soft palate, fauces, uvula, and pillars; Grade 2, visible soft palate, fauces, and uvula; Grade 3, visible soft palate and base of the uvula; and Grade 4, soft palate not visible. Grades 3 and 4 were high Mallampati classification which deemed a risk factor of hypoxemia during painless gastroscopy.

### Ultrasound protocol for tongue metrics assessment

**Figure 1:**
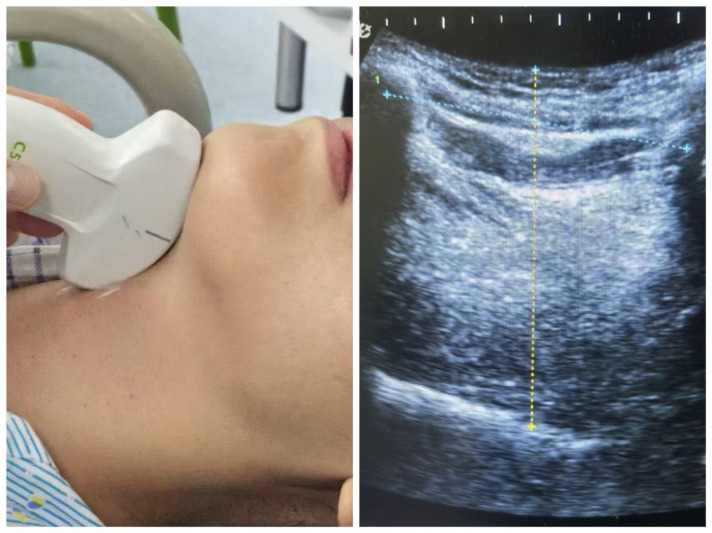
The method of tongue ultrasound measurement. (A) The low-frequency convex-array probe was placed to the skin of the neck in the midline in the submental sagittal plane; (B) The probe was adjusted to obtain entire tongue contour and the hyoid bone clearly on the screen. The TT of this patient is 62.4 mm and the hyomental distance is 53.1 mm as shown in the figure.

Prior to painless gastroscopy, all patients underwent sonographic measurement of tongue thickness (TT) and hyomental distance (HMD) in the outpatient waiting area using a low-frequency convex-array probe (Wisonic, Labat SP). With the patient in supine position and neck extended, standardized measurements were obtained under the following conditions: mouth closed with the tongue tip lightly touching the incisors, tongue fully relaxed without phonation. The transducer was positioned midline in the submental sagittal plane and carefully adjusted until the entire tongue contour was clearly visualized on the screen, at which point the image was frozen. Tongue thickness (TT) was measured as the maximal vertical distance from the tongue surface to the submental skin (Yao & Wang, 2017). The hyomental distance (HMD) was measured as the linear measurement between the hyoid bone and the inner cortex of the mandible (Wang et al., 2022) (Fig. 1).

All ultrasound examinations were performed consecutively and independently by two trained anesthesiologists, both of whom were experienced in comprehensive tongue ultrasonography. During the assessments, the operators were blinded to clinical information and followed identical standardized protocols during the same examination period. For each patient, the measurements (TT and HMD) were repeated three times, with the mean values calculated and recorded for subsequent analysis.

### Sedation protocol and painless gastroscopy procedure

In accordance with the standardized procedure (Zheng et al., 2023), all patients fasted for 8 h prior to the procedure and received 10 mL of dyclonine hydrochloride mucilage orally 30 min before examination. After establishing intravenous access in the forearm, patients were placed in the left lateral decubitus position and instructed on deep breathing techniques while receiving supplemental oxygen (three L/min) *via* nasal cannula with a bite-block in place. Continuous monitoring of heart rate (HR), blood pressure (BP), electrocardiogram (ECG), bispectral index (BIS), and pulse oximetry (SpO_2_) was maintained throughout the procedure. Following 1 min of preoxygenation, sedation was induced with propofol (two mg/kg) administered intravenously over 60 s by an anesthesiologist blinded to preoperative assessment results, followed by maintenance infusion at 2–5 mg/kg/h (Nonaka et al., 2015). The BIS was maintained between 50–65, with supplemental 0.5 mg/kg propofol boluses administered for BIS values >65 or signs of patient movement. All endoscopic procedures were performed by a single endoscopist. Upon completion, patients were transferred to the post-anesthesia care unit (PACU) under the supervision of an experienced anesthesia nurse for recovery monitoring.

### Data collection

The primary outcome was the incidence of hypoxemia (SpO_2_ < 90% for longer than 10 s). The secondary outcomes of the study were other adverse reactions (cough, body movements and hiccups) and severe adverse events. In this study, we defined severe adverse events as: moderate hypoxemia (Liu et al., 2018) (SpO_2_ < 85%), cough with hypoxemia, and prolapse of the bite block. Baseline characteristics included age, sex, underlying disease, body mass index (BMI), American Society of Anesthesiologists physical status (ASA) classification and the total dose of propofol.

In cases of hypoxemia occurring, immediate measures were taken including discontinuation of propofol administration, increasing oxygen flow rate from three to eight L/min, and performing jaw-thrust maneuver to maintain airway patency. If the SpO_2_ dropped below 85%, the endoscope was promptly withdrawn and mask ventilation was initiated. When SpO_2_ failed to improve within 1 min despite these interventions, endotracheal intubation was performed to establish mechanical ventilation (Vargo et al., 2009).

### Statistical analysis

Normality of data distribution was assessed using the Shapiro–Wilk test. The association between patient characteristics and hypoxemia occurrence was analyzed using appropriate methods based on data distribution characteristics: continuous variables with normal distribution were compared using Student’s *t*-test, non-normally distributed continuous variables were analyzed with the Mann–Whitney U test, while categorical variables were assessed using the *χ*2 test. The association between potential predictors and hypoxemia was evaluated using Spearman’s rank correlation coefficient. Initial screening of potential predictors was conducted through univariate logistic regression, with variables demonstrating marginal significance (*P* < 0.1) subsequently incorporated into multivariate logistic regression modeling. The final predictive model integrated both statistically significant covariates from the multivariate analysis and established risk factors documented in prior literature (Early et al., 2018). This composite model was visually represented through nomogram construction. The performance of the nomogram in terms of accuracy and stability was evaluated using receiver operating characteristic (ROC) curve analysis, calibration curve assessment, and decision curve analysis (DCA). All statistical analyses were conducted with R (version 4.4.2) and SPSS software version 24.0 (SPSS, Chicago, IL).

### Sample size

The sample size was calculated with the R software ver. 4.4.2. We conducted a pilot study for sample size assessment. The incidence of hypoxemia was 10.5%, a comparative analysis identified four statistically significant parameters. Subsequently, with the concordance index (C-index) set at 0.8, the calculated sample size was determined to be 293 cases, with 31 events of hypoxemia.

## Results

### General condition of patients

The study (*n* = 304) had a mean age of 50.4 years with 42.4% (129/304) male representation. Hypoxemia events (SpO_2_ < 90%) occurred in 32 patients (10.5%), with the majority (*n* = 28) resolving spontaneously or with simple interventions (chin lift or increased oxygen supplementation to six L/min *via* nasal cannula). A total of 11 patients developed moderate hypoxemia (SpO_2_ < 85%). Among them, four cases experienced severe desaturation (SpO_2_ < 70%) necessitating brief gastroscope removal and mask ventilation. Notably, all affected patients maintained sustained oxygenation recovery without requiring advanced airway management, enabling successful completion of the endoscopic procedures through timely clinical responses. Stratified analysis revealed significant between-group differences (hypoxemia *vs* non-hypoxemia) in anthropometric and airway parameters, including height, weight, body mass index, sonographic tongue thickness, hyomental distance, Mallampati classification, and total propofol dose. In contrast, demographic characteristics (age, gender) and ASA classification showed no statistically significant. Complete demographic characteristics and hypoxemia incidence data are presented in Table 1.

Regarding the other adverse events during the painless gastroscopy, a total of 44 patients (14.5%) developed cough reflex during sedation, with 10 cases (3.3% of total cohort) exhibiting concurrent hypoxemia (SpO_2_ < 90%). Involuntary body movements were observed in 33 patients (10.9%), including 17 instances (5.6%) of bite-block dislodgement requiring repositioning. Additionally, 23 patients (7.6%) experienced transient hiccups. All adverse events were successfully managed through temporary procedure suspension, supplemental propofol administration (0.3–0.5 mg/kg), or airway adjustment, with complete resolution achieved in all cases without requiring procedure termination or advanced airway intervention. There were no cases of pneumonia among these patients. Descriptive data of the adverse events of patients were shown in Table 2.

### Risk factors for hypoxemia during painless gastroscopy

Bivariate analysis revealed significant associations between hypoxemia occurrence and several predictive variables, as quantified by Spearman’s correlation coefficients: body mass index (*r* = 0.178, *p* = 0.002), tongue thickness (*r* = 0.279, *p* < 0.001), and Mallampati classification (*r* = 0.245, *p* < 0.001) demonstrated statistically significant positive correlations. While hyomental distance showed a weaker association (*r* = 0.108, *p* = 0.059) that did not reach conventional significance thresholds (Table 3).

**Table 1:** Comparison of baseline characteristics between the hypoxemia and the non-hypoxemia group.

Categorical variables	Hypoxemia	Non-hypoxemia	Test value	*P* value
		*n* = 272		
Sex (male/female, n)	17/15	112/160	1.673	0.296^a^
ASA classification(1/2/>2)	7/25/0	66/206/0	0.909	0.765^a^
Mallampati classification (>2/<3, n)	20/12	74/198	16.697	<0.001^a^

Continuous variables	Mean ± ss	Mean ± ss	t test	*P* value
Tongue thickness (mm)	61.6 ± 5.37	65.4 ± 6.31	−4.761	<0.001^b^
Hyomental distance (mm)	49.9 ± 6.37	47.2 ± 6.78	−2.146	0.033^b^
Total dose of propofol (mg)	165.2 ± 43.5	137.2 ± 28.9	−4.881	<0.001^b^
Age (y)	51 ± 14	50 ± 13	−0.378	0.705^b^
Height (cm)	166 ± 7	163 ± 7	−2.035	0.043^b^
Weight (kg)	69 ± 13	60 ± 11	−4.049	<0.001^b^
Body mass index (kg/m^2^)	24.8 ± 3.9	22.4 ± 4.1	−3.954	<0.001^b^

**Notes.**

Mean Average ss Standard Deviation

a Pearson Chi-Square.

b Independent sample t test.

**Table 2:** Categorization for adverse events and severe adverse events.

Adverse events		Severe adverse events
Hypoxemia (*n* = 32)		Moderate hypoxemia (*n* = 11)
Cough reflex (*n* = 44)		Cough hypoxemia (*n* = 10)
Body movement (*n* = 33)		Bite-block prolapse (*n* = 17)
Hiccups (*n* = 23)		/

**Notes.**

Cough hypoxemia cough with concurrent hypoxemia

**Table 3:** Correlation analysis of relevant predictive indicators and hypoxemia (*n* = 304).

Variable	r value	*P* value
BMI	0.178	0.002
Tongue thickness	0.279	<0.001
Hyomental distance	0.108	0.059
Mallampati classification	0.245	<0.001

**Notes.**

BMI body mass index

Logistic regression analysis was performed to identify the clinically significant risk factors for hypoxemia (Table 4). In univariate analysis, the risk of hypoxemia increased with BMI and propofol dose. In addition, the higher Mallampati classification and the higher tongue thickness, the higher the risk of hypoxemia. The gender, age, ASA classification and hyomental did not significantly increase the risk of hypoxemia. In the multivariate analysis, higher propofol dose, TT, and Mallampati classification were identified as independent risk factors for hypoxemia.

**Table 4:** Risk factors for hypoxemia during painless gastroscopy.

Univariate logistic regression (*n* = 304)			Multivariate logistic regression (*n* = 304)	
Variables	OR (95% CI)	*p* value	OR (95% CI)	*p* value
Gender	0.547 (0.240, 1.247)	0.151		
Age	1.010 (0.979, 1.042)	0.538		
ASA	1.420 (0.508, 3.971)	0.504		
BMI	1.270 (1.112, 1.452)	<0.001	1.084 (0.916, 1.282)	0.348
Propofol dose	1.028 (1.013, 1.043)	<0.001	1.020 (1.005, 1.035)	0.008
High M	4.868 (2.068, 11.459)	<0.001	3.370 (1.324, 8.579)	0.011
TT	1.177 (1.082, 1.281)	<0.001	1.120 (1.014, 1.238)	0.026
Hyomental	1.054 (0.988, 1.125)	0.111		

**Notes.**

ASA American society of anesthesiologists physical status classification system BMI body mass index High M Mallampati score >2 TT tongue thickness Hyomental hyomental distance OR odds ratio

### The nomogram to predict hypoxemia and predictive value

**Figure 2:**
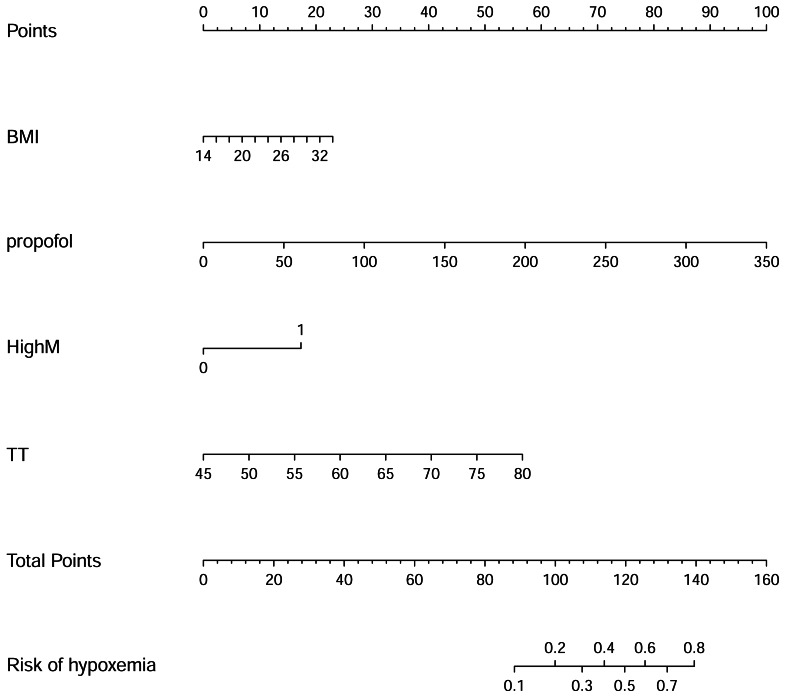
Nomogram for risk of hypoxemia under painless gastroscopy.

The nomogram to predict hypoxemia was created based on the following four independent factors (Fig. 2): BMI, propofol dose, high Mallampati score, TT. The higher total points obtained from the sum of the points for each factor in the nomogram means the greater possibility of hypoxemia during painless gastroscopy.

**Figure 3:**
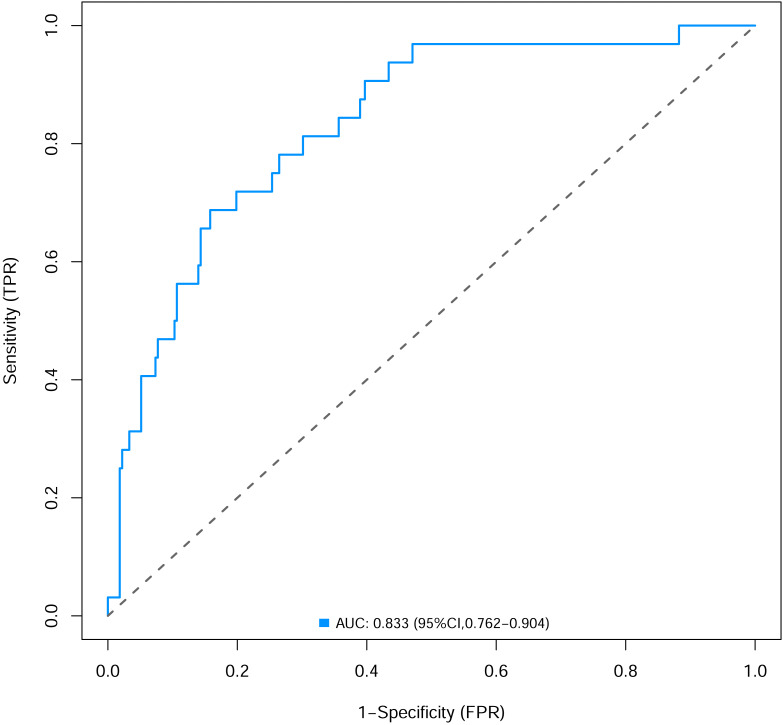
The areas under the receiver operating characteristic curves of nomogram.

**Figure 4:**
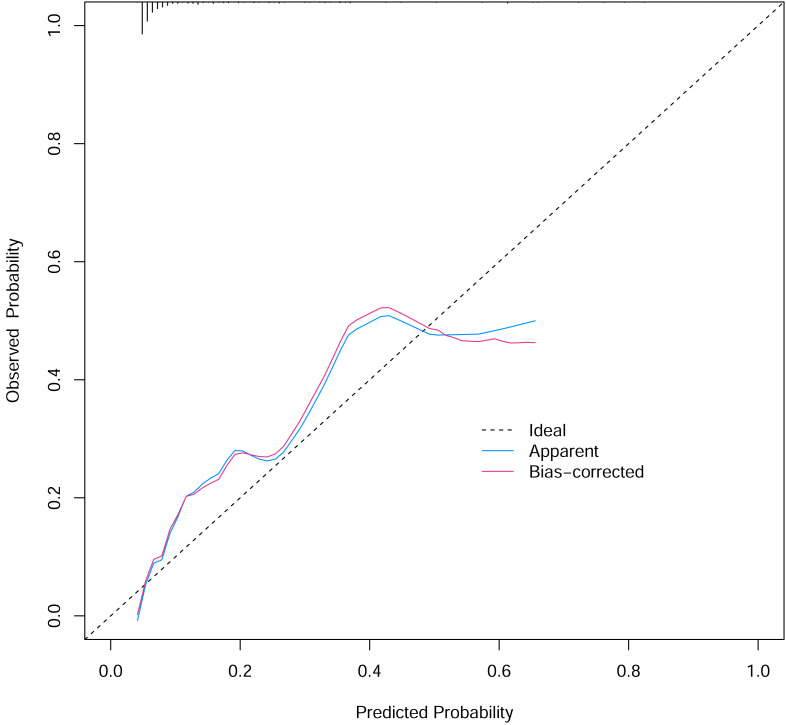
The calibration curve of the nomogram model.

**Figure 5:**
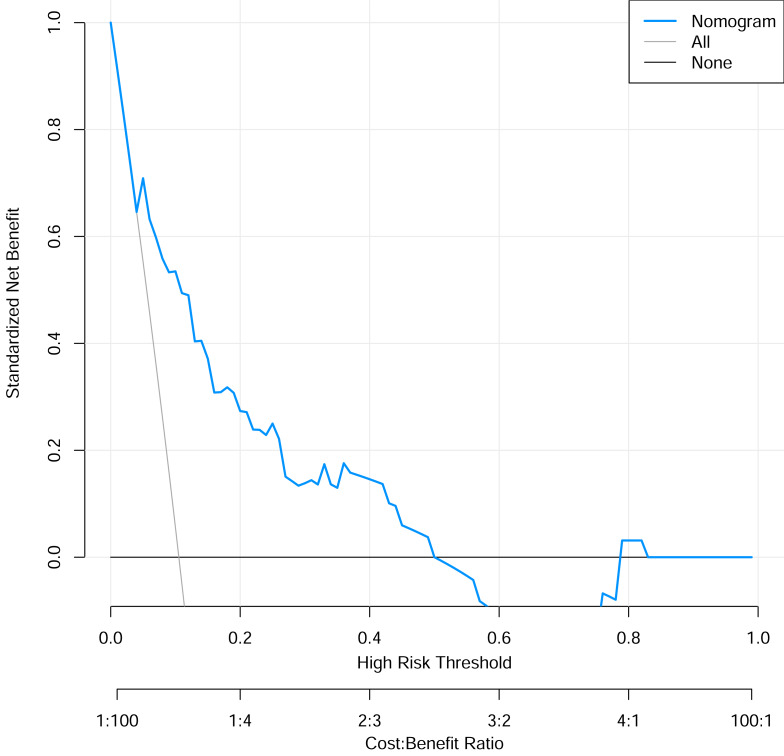
The decision curve analysis of the risk prediction nomogram model.

To verify the performance of the model, the ROC curve was constructed. The areas under the ROC curves were 0.833 (95% confidence interval (CI) [0.762–0.904]) (Fig. 3). To evaluate the model’s generalizability and mitigate overfitting, we employed K-fold cross-validation (*K* = 10). The dataset was randomly partitioned into 10 equal-sized subsets, with each subset serving as the validation set once while the remaining K−1 subsets were used for training. The model achieved an average area under the curve (AUC) of 0.801 (±0.03) across 10 folds. The Hosmer-Lemeshow test indicated good calibration of the nomogram, with no significant difference between predicted and observed risks (*χ*^2^ = 11.2, *p* = 0.191). The calibration curve analysis showed that the prediction probability of the nomogram model for pyrotinib-induced severe diarrhea was close to the actual probability, as shown in Fig. 4. The results of the decision curve analysis showed that the net benefit of the nomogram model was superior to both the all curve and none curve. Based on this decision curve, we can observe that within the threshold risk range of 0%–50%, intervention decisions based on the predictive model are clearly beneficial (Fig. 5).

## Discussion

In this study, we identified tongue thickness measured by ultrasonography as an independent risk factor for hypoxemia during painless gastroscopy, which aligns with previous findings that tongue thickness serves as a critical predictor of difficult airway management (Ovassapian et al., 2002).

Adequate preoperative evaluation remains crucial for optimizing patient condition, improving clinical outcomes, and enhancing comfort. Currently, there is no internationally standardized preoperative assessment protocol for painless gastrointestinal endoscopy, nor has a comprehensive and rational standardized procedure been established (Xu et al., 2024). Both the American Society of Anesthesiologists (ASA) Physical Status Classification (Early et al., 2018) and the 2021 Korean Guidelines (Park et al., 2022) for Endoscopic Sedation utilize the ASA classification to evaluate the risk of painless gastroscopy, which also serves as the primary assessment tool in most domestic hospitals. Although the ASA classification demonstrates broad applicability and correlates with perioperative cardiopulmonary adverse events, it lacks the granularity required for individualized risk assessment. Establishing a more robust evaluation framework is therefore necessary to mitigate procedure-specific complications associated with various gastrointestinal endoscopic techniques. Such a system should integrate multiple validated risk assessment tools to support comprehensive preoperative evaluation. This study represents a preliminary exploration in the local population. By incorporating ultrasonography—a commonly used visualization technique—into the assessment, the resulting predictive model may possess certain potential for broader application.

During painless gastroscopy, intravenous anesthesia often induces tongue base retraction, which obstructs the pharyngeal space (Razavi, Gross & Katz, 2014). Combined with upper airway collapse and respiratory depression, this mechanism may collectively contribute to the occurrence of hypoxemia. Consequently, greater tongue thickness results in more severe oropharyngeal narrowing during tongue base retraction in painless gastroscopy, leading to a higher incidence of hypoxemia. In our study, the hypoxemia group demonstrated significantly greater tongue thickness than the non-hypoxemia group (*p* < 0.001), with multivariate regression analysis confirming tongue thickness as an independent predictor (odds ratio (OR) = 1.12, 95% CI [1.014–1.238], *p* < 0.05). However, the hyomental distance (a surrogate marker of tongue length) showed no significant correlation with hypoxemia in either univariate or multivariate analyses (*p* > 0.05). This phenomenon may primarily stem from the partial overlap—yet distinct differences—between difficult airway scenarios and hypoxemia mechanisms during painless gastroscopy. For instance, increased tongue thickness negatively correlates with laryngoscopic view quality and subsequently elevates intubation difficulty (Ovassapian et al., 2002)—a pattern mechanistically analogous to how greater tongue thickness exacerbates oropharyngeal narrowing during tongue base retraction, explaining the consistent findings. In contrast, while reduced hyomental distance increases laryngoscope curvature relative to airway anatomy (Gomes et al., 2021) (compromising optimal tip positioning and glottic visualization for intubation), this anatomical relationship does not contribute to hypoxemia pathogenesis during gastroscopy.

High BMI and large dosage of sedatives are independent risk factors for hypoxemia during painless gastroscopy (Friedrich-Rust et al., 2014). In our study, the total dose of propofol was identified as an independent risk factor for hypoxemia, a finding consistently supported by both intergroup comparisons and univariate and multivariate regression analyses. During painless gastroscopy, the administration of propofol is determined by multiple factors: weight, target bispectral index values, and the occurrence of adverse events such as involuntary body movements, which may necessitate additional doses. Furthermore, procedural duration can influence the total propofol requirement. Although the total propofol dose cannot be predicted prior to the procedure, we included it as a predictive parameter in our nomogram due to its potential utility in anticipating hypoxemia during the post-procedural recovery phase. For instance, patients requiring high propofol doses exhibit an elevated risk of hypoxemia not only intraoperatively but also postoperatively in the recovery unit. Thus, monitoring propofol dosage provides clinically actionable predictive value, enabling anesthesiologists to implement proactive measures to mitigate hypoxemia risk. Multivariate regression analysis in this study did not reveal a statistically significant association between BMI and hypoxemia (*p* > 0.05). We speculate that this may be attributed to the relatively small number of overweight/obese patients (high BMI) included in our cohort. This limitation likely stems from the stringent preoperative anesthesia assessment in our clinic, where high-risk patients were counseled on potential complications and subsequently opted for conventional gastroscopy instead. Nevertheless, given the well-established role of elevated BMI as an independent risk factor for hypoxemia in prior studies (Geng et al., 2019; Wang et al., 2023), we retained it as a key predictive variable in our nomogram to ensure clinical generalizability and alignment with existing evidence.

In this study, 11 patients experienced moderate hypoxemia (SpO_2_ < 85%), all of which were alleviated after timely intervention. Due to the small sample size (*n* = 11), effective analysis of the underlying causes was not feasible. Future studies with larger sample sizes may yield more meaningful insights. Additionally, 44 patients exhibited a cough reflex during the procedure, among whom 10 developed hypoxemia following coughing, possibly due to reflux aspiration (Bielawska et al., 2018). Given the limited number of positive events (coughing accompanied by hypoxemia), future studies with expanded sample sizes are warranted to analyze potential risk factors. Furthermore, 17 patients experienced involuntary body movements that led to the dislodgement of the endoscope mouthpiece, all occurring during the transition from consciousness to anesthesia. This complication resulted in clenched jaws, biting of the endoscopic equipment, and difficulty in reinserting the mouthpiece. The causes of such involuntary movements and preventive strategies merit further investigation. In this study, 23 patients (7.6%) developed hiccups, a lower incidence compared to the 20.5% reported in the study of Liu et al. (2012). The primary reason may be that this study exclusively used propofol for sedation, without combining other medications known to increase the risk of hiccups. Although hiccups can interfere with the procedure and potentially elevate the risks of reflux aspiration and hypoxemia (Steger, Schneemann & Fox, 2015), all observed cases in this study were short-lived and resolved after deepening anesthesia, with no subsequent adverse effects reported.

It is noteworthy that a recent study developing a prediction model for hypoxemia in middle-aged and elderly Chinese outpatients undergoing painless gastroscopy demonstrated that the random forest model, among machine learning algorithms, exhibited optimal predictive performance (Zheng et al., 2025). That study identified BMI and anesthetic dosage as risk factors for hypoxemia, which aligns with our findings and further substantiates the rationale and necessity of including BMI in the construction of our nomogram. In our nomogram models, the hypoxemia prediction nomogram incorporated four variables (BMI, propofol dose, high Mallampati classification, TT). Among these variables, BMI, propofol dose, high Mallampati classification were traditional hypoxemia assessment methods while TT were ultrasonography-assisted assessment methods. The prediction model integrating ultrasonography as a visualization technique with conventional predictive indicators demonstrates significant advantages. The primary rationale lies in the capability of ultrasound to identify upper airway anatomical landmarks (Singh et al., 2010; Lahav et al., 2009; Xia et al., 2011), a feature unattainable by traditional external anatomical landmark-based parameters. The nomograms in this study are easy to implement in routinely clinical practice.

In recent years, ultrasound technology has gained widespread adoption within anesthesiology departments, and proficiency among anesthesiologists in using this visual tool has become increasingly common (*e.g.*, for difficult airway assessment (Lin et al., 2023), gastric content evaluation (Evain et al., 2022), and pre-procedural neuraxial imaging (Kamimura et al., 2024)). Therefore, the feasibility of implementing this ultrasound assessment is high for anesthesiologists. As our results show, the prediction model we constructed achieved an AUC of 0.833, indicating considerable predictive power for hypoxemia risk. This high predictive value is critically important. It means that if a patient is identified as being at high risk for hypoxemia, detailed preventive plans can be activated in advance. This includes preparing specific equipment like endoscopy-specific airways or opting for conventional gastroscopy, thereby significantly enhancing patient safety and preventing adverse outcomes. The clinical benefit derived from proactively avoiding such incidents is substantial.

The new knowledge generated by this study primarily includes: firstly, to our knowledge, it is the first study to develop and validate a practical nomogram that integrates a novel, objective sonographic metric (tongue thickness) with conventional risk factors for predicting hypoxemia specifically in patients undergoing painless gastroscopy. Secondly, our findings elucidate a distinct mechanism for hypoxemia in this setting, confirming the critical role of tongue thickness while demonstrating that hyomental distance may be less relevant, which refines the current understanding of airway risk assessment during procedural sedation. This study supports the integration of tongue ultrasonography into the standard preoperative workflow in the anesthesia clinic for ASA I-II patients undergoing painless gastroscopy. We propose a clinical pathway whereby sonographic tongue thickness, combined with BMI and Mallampati score, is input into our nomogram to generate a quantifiable hypoxemia risk score. This score facilitates immediate risk stratification. High-risk patients, thus identified, may benefit from a bundle of preventive interventions, such as the utilization of endoscopy-specific airways, management by an experienced anesthesiologist, pre-emptive setup of nasopharyngeal airways, or consideration of conventional gastroscopy as a safer alternative.

This study has some limitations. Firstly, the single-center design and relatively limited number of hypoxemia cases may affect the generalizability of our findings. Secondly, the study population consisted exclusively of ASA physical status I-II patients who underwent preprocedural anesthetic evaluation, resulting in underrepresentation of obese individuals—a phenomenon that may be partially attributed to anthropometric differences between Asian and Caucasian populations. Thirdly, despite our efforts to adjust for known confounders through multivariate analysis, the possibility of residual confounding cannot be entirely ruled out. Unmeasured variables, such as undiagnosed obstructive sleep apnea (OSA), detailed pulmonary function, smoking history, the complexity of the examination procedure, or neck circumference, might also influence the risk of hypoxemia and were not accounted for in our model. These factors are associated with the outcome of the study. We will expand our research to include larger sample sizes and investigate these factors in the future. Finally, although propofol remains the fundamental agent for sedation in painless gastrointestinal endoscopy (Song & Peng, 2024), newer anesthetic agents such as remimazolam and ciprofol are increasingly being adopted in painless gastroscopy practice (Ye et al., 2023; Shan et al., 2024). Future studies should address these limitations. Broader implementation would require multicenter studies and confirmation in higher-risk populations.

## Conclusions

The thickness of the tongue, as measured by ultrasound, has been identified as an independent risk factor for hypoxemia during painless gastroscopy. The nomogram incorporating tongue thickness, Mallampati score, BMI, and total propofol dosage demonstrates important predictive potential. This tool holds promise for offering valuable guidance and reference in the pre-procedural assessment in painless gastroscopic examinations.

##  Supplemental Information

10.7717/peerj.20634/supp-1 Supplemental Information 1 Raw data

10.7717/peerj.20634/supp-2 Supplemental Information 2 Codebook for raw data

10.7717/peerj.20634/supp-3 Supplemental Information 3 STARD checklist

10.7717/peerj.20634/supp-4 Supplemental Information 4 rebuttal letter for author

10.7717/peerj.20634/supp-5 Supplemental Information 5 photo permission letter

## Additional Information and Declarations

### Data Availability

The following information was supplied regarding data availability: The raw measurements are available in the Supplemental File.

### Clinical Trial Registration

The following information was supplied regarding Clinical Trial registration: ChiCTR2000032940.
