# Supplementary material for: Development of a tongue ultrasound-based predictive model for hypoxemia during painless gastroscopy in ASA I-II patients
Source: PeerJ. 2026 Jan 14;14:e20634. doi: 10.7717/peerj.20634 (PMC12811964; doi:10.7717/peerj.20634)
Supplement: Supplemental Information 2 [file peerj-14-20634-s002.docx]

| **Variable** | **Number code** | **meaning** |
| --- | --- | --- |
| gender | 1 | Male |
| gender | 2 | Female |
| obesity | 0 | Not obesity |
| obesity | 1 | obesity |
| Hypoxemia | 0 | Not hypoxemia |
| Hypoxemia | 1 | hypoxemia |
| Severe Hypoxemia | 0 | Not |
| Severe Hypoxemia | 1 | Severe Hypoxemia |
| Cough reflex | 0 | Not |
| Cough reflex | 1 | Cough reflex |
| Hiccups | 0 | Not |
| Hiccups | 1 | Hiccups |
| bodymove | 0 | Not |
| bodymove | 1 | bodymove |
| M | / | Mallampati classification |
| High M | 0 | M＜3 |
| High M | 1 | M＞2 |
