# Supplementary material for: Development of a tongue ultrasound-based predictive model for hypoxemia during painless gastroscopy in ASA I-II patients
Source: PeerJ. 2026 Jan 14;14:e20634. doi: 10.7717/peerj.20634 (PMC12811964; doi:10.7717/peerj.20634)
Supplement: Supplemental Information 5 [file peerj-14-20634-s005.docx]

**PeerJ Photo/Video Permission Letter**

1. **REQUIRED** Title of PeerJ submission: **Development of a tongue ultrasound-based predictive model for hypoxemia during painless gastroscopy in ASA I-II patients**
2. **REQUIRED** Printed name of copyright holder (must include the name of an individual and their title when signing on behalf of a company/institution. Company/institution names alone are not acceptable): **Chen Gao (高晨)**
3. **REQUIRED** *For figures*: Paste the approved images(s) including the figure number(s) as they appear in the PeerJ submission below *For* *videos*: Please supply a brief description of the video(s) including the title, description of content, length of video
   Use additional pages as necessary for multiple figures / videos

| 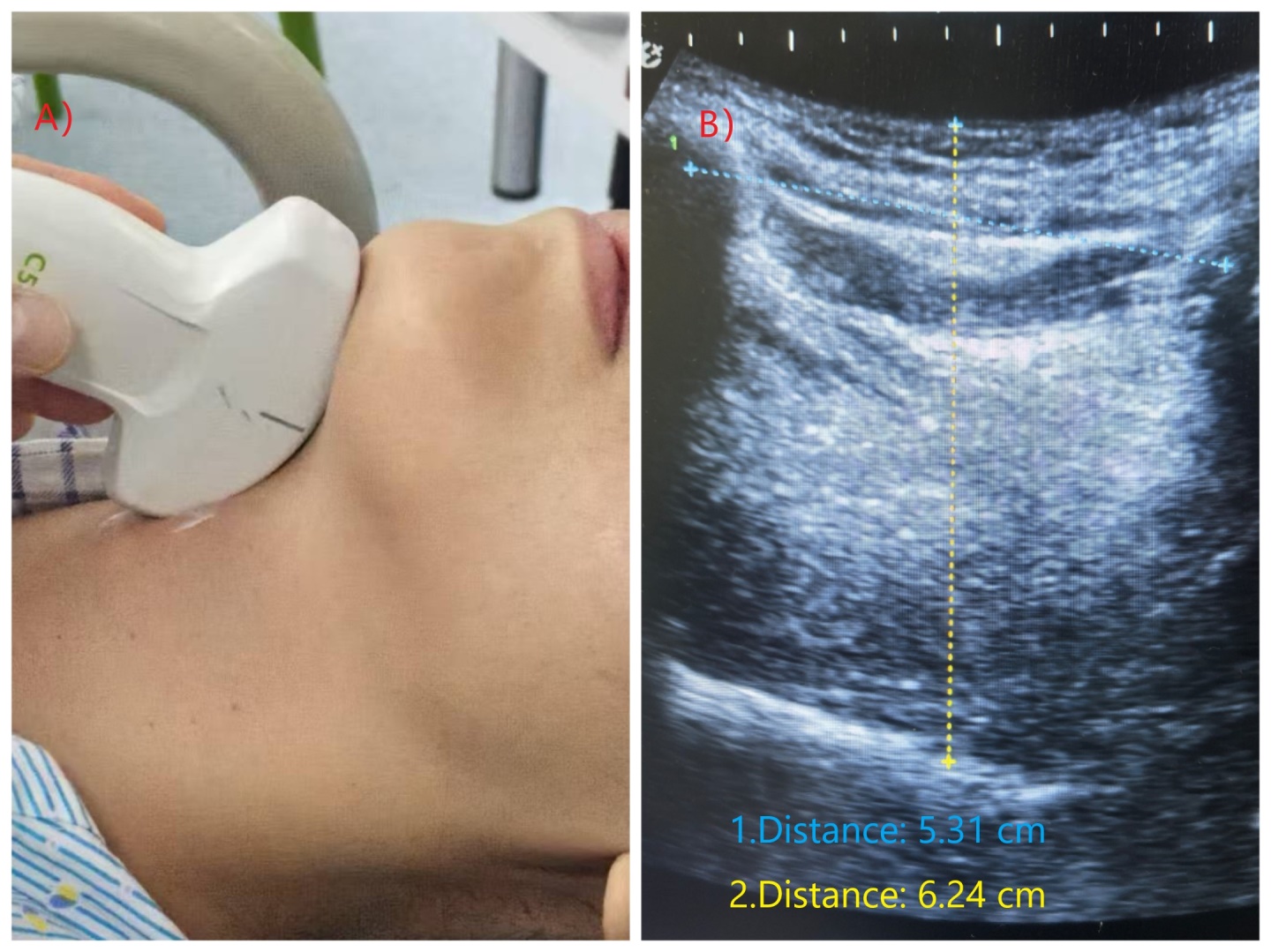  **Figure 1** |
| --- |

I give my permission to PeerJ to publish the work, as appears above, under the CC-BY 4.0 license (https://peerj.com/about/policies-and-procedures/#open-access-copyright-policy).

**REQUIRED *Signature of copyright holder***
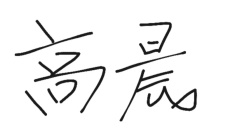
_
