# Supplementary material for: Development of a tongue ultrasound-based predictive model for hypoxemia during painless gastroscopy in ASA I-II patients
Source: PeerJ. 2026 Jan 14;14:e20634. doi: 10.7717/peerj.20634 (PMC12811964; doi:10.7717/peerj.20634)
Supplement: Supplemental Information 4 [file peerj-14-20634-s004.docx]

**Rebuttal: Justification for Addition of New Authors**

Dear Editors and Reviewers,

This letter aims to provide the required detailed justification and contribution explanation regarding the addition of new authors in the revised version of our manuscript. We fully understand the journal's strict policies regarding changes in authorship and confirm that all authors, including the newly added ones, have been informed and have consented to this change.

**1. Justification for Adding New Authors**

The following new author has been added to this revised version:

**Name:** Xu Chen

**Affiliation:** Department of Anesthesiology, The First Affiliated Hospital of USTC, Division of Life Sciences and Medicine, University of Science and Technology of China

**Justification:** Dr. Xu Chen has been a valuable member of our research team. Prior to the formal commencement of this study, specifically before its successful registration with the Chinese Clinical Trial Registry, he participated in preliminary design, planning, and preparatory work related to this research. Following the initiation of the study, he was involved in the ultrasonography measurements within the trial methodology and also contributed to addressing the reviewers' comments, data collection, and statistical analysis. These contributions are critical components of this manuscript. Hence, his contributions fully meet the authorship criteria defined by the ICMJE.

**2. Details of New Author's Specific Contributions**

The following is a detailed breakdown of the specific contributions made by the newly added author, Xu Chen, to this manuscript:

| Contribution Category | Specific Description |
| --- | --- |
| **Research Conception and Design** | Participated in designing the experimental protocols. In particular, his familiarity with perioperative point-of-care ultrasound examination techniques significantly contributed to the refinement of our preliminary design concepts. |
| **Data Acquisition** | Was responsible for the collection and documentation of a portion of the experimental data, related statistical analyses. |
| **Data Analysis and Interpretation** | Conducted professional statistical analysis of the obtained data and co-wrote the relevant sections of the Results. |
| **Manuscript Drafting and Revision** | Drafted a portion of the initial version of the manuscript. Also reviewed and provided critical revisions to the scientific rigor of the entire manuscript. |

**3. Explanation Regarding Omission in Initial Submission**

As mentioned in our initial response to your letter, Dr. Xu Chen was originally included in the initial author list. However, due to exceptional circumstances (a malfunction in his email system that prevented login access), he was unable to complete the verification process and could not be contacted. To ensure timely submission of the manuscript, we proceeded with the submission while omitting his authorship information. After his email system was restored to normal operation and he became able to confirm his authorship, he has now been formally added to the author list. We hereby formally submit this request and would like to express our sincere apologies for this oversight.

4**. Confirmation**

We hereby confirm that all authors, including the newly added author Xu Chen and all original authors, have formally approved this change to the author list by responding to the email confirmation links. We understand and agree that this represents the final author list for the manuscript and that no further changes will be accepted.

Thank you for handling our manuscript and considering this request.

Sincerely,

Lijian Chen
